# Supplementary material for: Seasonal Changes in Serum Metabolites in Multiple Sclerosis Relapse
Source: Int J Mol Sci. 2023 Feb 10;24(4):3542. doi: 10.3390/ijms24043542 (PMC9959388; doi:10.3390/ijms24043542)
Supplement: Supplementary file 1 [file ijms-24-03542-s001.zip › ijms-2153898-supplementary.pdf]

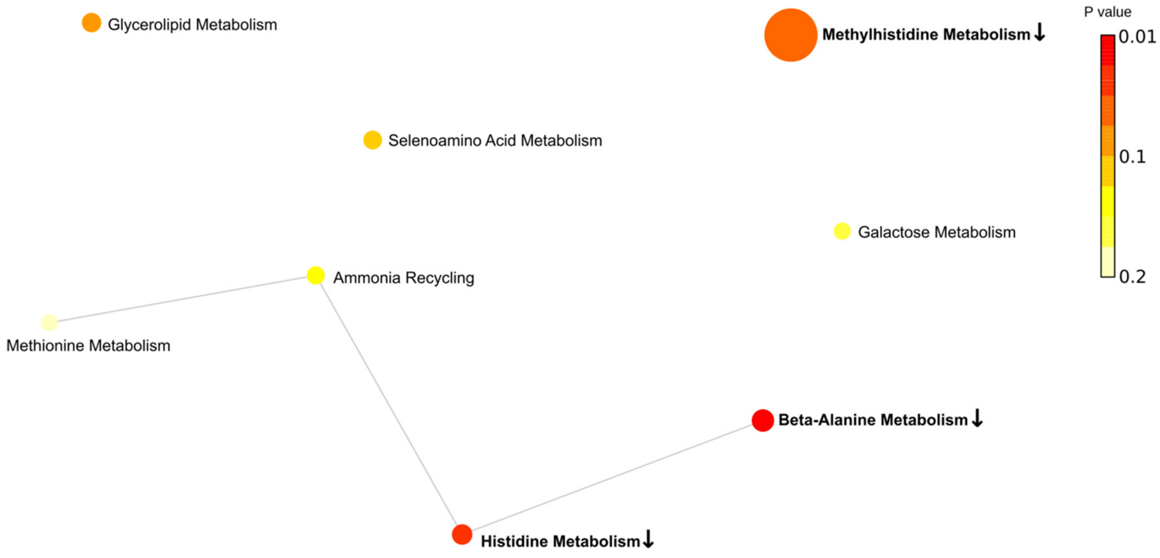

Figure S1. Metabolic pathways affected both spring and fall MS. Bold indicates significantly enriched pathways ($p < 0.05$). ↓—decreased serum level of metabolites included in the metabolic pathway in MS compared to control. Each node represents a metabolite set with its color based on its *p* value and its size based on fold enrichment. Two metabolite sets are connected by an edge if the number of their shared metabolites is over 25% of the total number of their combined metabolite sets. The yellow to red color gradient and larger size of the circle indicate lower *p* value.

Table S1. List of metabolites significantly changed in MS group compared to controls.

	MS fall vs. CTRL fall		MS winter vs. CTRL winter		MS spring vs. CTRL spring		MS summer vs. CTRL summer	
	**Decreased (59)**	**Increased (6)**	**Decreased (12)**	**Increased (18)**	**Decreased (21)**	**Upregulated (7)**	**Decreased (10)**	**Increased (8)**
1	Coenzyme Q9	Ceramide(d18:1/16:0)	DHC(18:1/24:0)*	Ceramide(d18:1/22:0 OH)	DHC(18:1/24:0)*	Ceramide(d18:1/24:0)	DHC(18:1/24:0)*	Ceramide(d18:1/22:0 OH)
2	Cer(d18:1/12:0)	Ceramide(d18:1/18:1 OH)	Vitamin D3	Ceramide(d18:1/24:0)	Vitamin D3	Ceramide(d18:1/16:0)	Vitamin D3	Ceramide(d18:1/24:0)
3	PG (16:0/16:0)	Ceramide(d18:1/18:1)	N-Acetylserine	Ceramide(d18:1/16:0)	Riboflavin	Ceramide(d18:1/18:1 OH)	Hexacosanoic acid*	Ceramide(d18:1/18:1 OH)
4	DHC(18:1/24:0)*	Niacinamide	5-Hydroxy-L-tryptophan	Cer(d18:1/12:0)	Selenomethionine	Cytidine monophosphate	L-Lactic acid	Cytidine monophosphate
5	Ureidopropionic acid	Sphingosine*	Hexacosanoic acid*	Ceramide(d18:1/18:1)	Chenodeoxycholic acid	Sphingosine*	Gentisic acid*	Sphingosine*
6	2 Aminocaprylic acid	Deoxyguanosine 5′-monophosphate	4,5-Dihydroorotic acid	Glucosamine	Hexacosanoic acid*	Deoxyguanosine	O-Phosphorylethanolamine	L-Arginine
7	N-Acetylserine		Allantoin	Alpha-Tocopherol	Allantoin	Deoxyguanosine 5′-monophosphate	CDP-choline	Deoxyguanosine
8	S-Adenosylhomocysteine		L-Lactic acid	Biotin	4-Aminohippuric acid		Docosahexaenoic acid(DHA)	Guanosine
9	L-Arginine		4-Hydroxyphenylpyruvic acid	Sphingosine*	Agmatine		DL-O-Phosphoserine	
10	L-Asparagine		Gentisic acid*	Eicosapentaenoic acid	Glycerol		Gamma-Aminobutyric acid	
11	Selenomethionine		Glycylproline	Linoleic acid	Gentisic acid*			

12	PE(18:0/16:1(9Z))	Methylguanidine	Deoxyguanosine	Plasmalogen(p18:0/22:6)
13	Hexacosanoic acid*		Quinolinic acid	LysoPE(P-18:0/0:0)
14	4,5-Dihydroorotic acid		Homovanillic acid	Docosahexaenoic acid(DHA)
15	Glycolic acid		N-Methyltyramine	Hydroxykynurenine
16	Anandamide		2-Arachidonylglycerol	Glycylproline
17	4-Hydroxyphenylpyruvic acid		PE(20:1(11Z)/16:0)	Methionine sulfoxide
18	Glyceric acid		PC(18:0/18:2(9Z,12Z))	Gamma-Aminobutyric acid
19	N-Acetylputrescine			Carnosine
20	4-Aminohippuric acid			L-Histidine
21	Agmatine			Methylguanidine
22	Homovanillic acid			
23	Mevalonic acid			
24	Glycerol			
25	Normetanephrine			
26	Glucose			
27	Myo-Inositol			
28	Gluconolactone			
29	Glucose 6-phosphate			
30	Ascorbic acid			
31	Gentisic acid*			
32	2-Keto-L-gluconate			
33	N-a-Acetyl-L-arginine			
34	Plasmalogen(p18:0/22:6)			
35	LysoPE(P-18:0/0:0)			
36	CDP-choline			
37	Docosahexaenoic acid(DHA)			
38	L-Homoserine			
39	Hydroxykynurenine			
40	L-Phenylalanine			
41	L-Leucine			
42	L-Isoleucine			
43	L-Valine			
44	Glycylproline			
45	Methionine sulfoxide			
46	O-Acetylserine			
47	Dimethyl-L-arginine			
48	Gamma-Aminobutyric acid			
49	L-Asparagine			
50	Homocysteine			
51	Methylcysteine			
52	Ornithine			
53	L-Kynurenine			
54	Carnosine			
55	L-Histidine			
56	L-Glutamic acid			
57	L-Cystathionine			

58	Hypotaurine
59	Picolinic acid
